# Advances in the understanding of IgM monoclonal gammopathy of undetermined significance

**DOI:** 10.12688/f1000research.12880.1

**Published:** 2017-12-18

**Authors:** Jonas Paludo, Stephen M Ansell

**Affiliations:** 1 Department of Medicine, Division of Hematology, Mayo Clinic , Rochester, USA

**Keywords:** IgM gammopathy, MGUS, Waldenstrom macroglobulinemia

## Abstract

Among monoclonal gammopathies of undetermined significance (MGUSs), the immunoglobulin M (IgM) MGUS subtype stands as a unique entity and plays a pivotal role as a pre-malignant condition for multiple B-cell non-Hodgkin lymphomas, most notably Waldenström macroglobulinemia (WM). A relationship between IgM MGUS and WM has been proposed for decades. However, insight regarding the pathobiology of these two conditions improved significantly in recent years, strengthening the hypothesis that WM and IgM MGUS are different stages of the same disease. Therefore, the understanding of IgM MGUS and that of WM are interconnected and advances in one will likely impact the other. Furthermore, IgM MGUS has been more commonly recognized as the underlying etiology of IgM-related disorders. In this review, we explore recent advances in the understanding of the pathobiology of IgM MGUS and WM and the treatment of common IgM-related disorders.

## Introduction

Even though multiple myeloma (MM), chronic lymphocytic leukemia (CLL), and Waldenström macroglobulinemia (WM) are unique diseases with distinct presentations, prognoses, and treatment strategies, they all originate from B cells in the late stages of differentiation ^1^. CLL arises from a pre- or post-germinal center CD5 ^+^ B cell ^2^. WM arises from a post-germinal center B cell that has undergone somatic hypermutation but not heavy-chain class switch ^3^. The cell of origin in MM is a B cell that has fully differentiated into a plasma cell ^4^.

Along with the closely related cells of origin, these diseases also seem to be preceded by pre-malignant conditions. CLL and MM are always preceded by monoclonal B-cell lymphocytosis (MBL) and monoclonal gammopathy of undetermined significance (MGUS), respectively ^5, 6^. Although immunoglobulin M (IgM) MGUS is the probable precursor condition for WM, the relationship between these two entities is not as well stablished as in CLL and MM. Moreover, IgM MGUS has been shown to be a distinct entity from non-IgM or light-chain MGUS ^7^.

This review explores recent advances in the understanding of IgM MGUS and its relationship with WM and focuses on the pathobiology and management of IgM MGUS.

## IgM MGUS

The definition of IgM MGUS is not universally agreed upon. The Mayo Clinic criteria characterize IgM MGUS as a serum IgM monoclonal protein of less than 3 g/dL, bone marrow lymphoplasmacytic lymphoma (LPL) involvement of less than 10%, and no evidence of signs or symptoms secondary to the lymphoplasmacytic infiltrative process ^8^. In contrast, the Second International Workshop on WM consensus defined IgM MGUS as a serum IgM monoclonal protein of any size without bone marrow involvement by an LPL ^9^. A monoclonal IgM paraprotein can be detected in a number of diseases, and a differential diagnosis of IgM monoclonal gammopathies is shown in Table 1.

**Table 1.** Primary differential diagnoses of IgM monoclonal gammopathies.

	IgM MGUS	Smoldering/ asymptomatic WM	WM	IgM multiple myeloma	IgM amyloidosis	Splenic marginal zone lymphoma
Serum IgM gammopathy	<3 g/dL	≥3 g/dL	Any level	Any level	Any level	Low level
Bone marrow LPL infiltrate, percentage	<10%	≥10%	≥10%	≥10%; predominantly plasmacytic PCs	Normal or slight increase of PC or LPL	Intertrabecular and intrasinusoidal infiltrate
End-organ damage/ Symptoms	No	No	Yes ^a^	Yes ^b^	Yes ^c^	Yes ^d^
Hyperviscosity	No	No	Yes	Uncommon	Uncommon	Uncommon
Differentiating genetic features and markers	6q deletion absent, MYD88 L265P (up to 80%)	6q deletion, MYD88 L265P (90%), CD56 ^−^	6q deletion (30–50%), IgH translocations absent, MYD88 L265P (90%), CD56 ^−^, CD25 ^+^ (88%), CD103 ^−^	May have t(11;14) or other IgH translocations, MYD88 L265P- negative CD56 ^+^, CD138 ^+^ CD19 ^−^, CD 45 ^−^	May have t(11;14)	+3q (19%) and +5q (10%); MYD88 L265P-negative CD 22 ^+^, CD11c ^+^, CD25 ^−^, Del 7q (19%), CD25 ^+^ (44%), CD103 ^+^ (40%)

The table lists a few important differential diagnoses of immunoglobulin M (IgM) monoclonal gammopathies. IgM paraprotein can be present in virtually all B-cell lymphoproliferative disorders. ^a^Constitutional symptoms: hepatosplenomegaly, lymphadenopathy, anemia, hyperviscosity, solid organ involvement, and rarely lytic lesions. ^b^CRAB (hypercalcemia, renal failure, anemia, and bone lesions) features. ^c^Organs typically involved are kidneys, heart, nerves, tongue, gastrointestinal tract, and liver. Patients with IgM amyloid light-chain (AL) amyloidosis have higher frequency of pulmonary, lymph node, peripheral nerve, and lower cardiac involvement. Concentration of free light-chain tends to be lower compared with non-IgM AL amyloidosis. ^d^Primarily involves spleen; lymphadenopathy is rare. LPL, lymphoplasmacytic lymphoma; MGUS, monoclonal gammopathy of undetermined significance; PC, plasma cell; WM, Waldenström macroglobulinemia. Reprinted from Blood Reviews, Vol 29/Issue 5, Prashant Kapoor,Jonas Paludo,Nishanth Vallumsetla,Philip R. Greipp, Waldenström macroglobulinemia: What a hematologist needs to know, page 301-319, DOI: 10.1016/j.blre.2015.03.001, Copyright (2015), with permission from Elsevier ^42^.

The prevalence of MGUS, based on large population studies, is estimated at 2 to 3% in adults over the age of 50 years, and the median age at diagnosis is about 70 years. The incidence of MGUS increases with age and is more common in males than females. IgM MGUS represents only 15 to 17% of all MGUS cases ^10, 11^. Whereas the prevalence of MGUS seems higher in African-Americans (3.7%) and African blacks (5.8%) compared with Caucasians (2.3%), the prevalence of the IgM subtype is fivefold higher in Caucasians compared with African-Americans ^11, 12^. This racial disparity is consistent with the reported higher incidence of WM in Caucasians ^13^.

Patients with IgM MGUS are at increased risk of progression to a hematologic malignancy. Long-term follow-up of 213 patients with IgM MGUS showed the development of non-Hodgkin lymphoma (NHL) in 17 patients, WM in six patients, CLL in three patients, and light-chain (AL) amyloidosis in three patients. The risk of developing any lymphoid neoplasm or related disorder compared with the expected incidence was 16-fold higher in patients with IgM MGUS. The risk of developing WM was 262-fold higher. The probability of progression was estimated at 1.5% per year and the risk continued to increase even 20 years after the initial diagnosis ^8^. The initial concentration of M-protein at IgM MGUS diagnosis is a strong predictor for progression. Patients with an M-protein of more than 2.5 g/dL had a 3.1-fold greater risk for progression than those with an M-protein of less than 0.5 g/dL, likely reflecting the impact of a higher tumor burden on the risk of progression ^14^. Early studies have also suggested that IgM MGUS patients harboring the MYD88 ^L265P^ mutation have an increased risk of progression to WM ^15, 16^. However, the pathogenic role of the MYD88 ^L265P^ mutation in the transformation to WM has not been established yet. The relevance of IgM MGUS lies not only in the increased risk of progression to a hematologic malignancy but also in the risk of developing end-organ damage secondary to autoimmune properties or deposition of the IgM paraprotein ^9^.

## Pathobiology of IgM MGUS and Waldenström macroglobulinemia

The understanding of the pathobiology of IgM MGUS and WM has grown significantly over the last few years as technological advances, such as multidimensional flow cytometry (MFC), gene expression profiling (GEP), and next-generation sequencing (NGS), provided valuable insight into the unique relationship between these two entities. LPL, an indolent NHL, is characterized by bone marrow infiltration with malignant cells ranging from small mature-appearing B cells to lymphoplasmocytes and plasma cells ^17^. Presence of LPL is a requirement for the diagnosis of WM but can also be identified in patients with IgM MGUS depending on the diagnostic criteria used ^9, 14^. A large cohort of IgM MGUS patients with long-term follow-up seen at Mayo Clinic demonstrated less than 10% bone marrow involvement by an LPL in all assessed patients and influenced the current Mayo Clinic diagnostic criteria for IgM MGUS ^14^. Additionally, bone marrow evaluation by MFC of patients with IgM MGUS showed less than 10% bone marrow infiltration by clonal B cells in 99% of the cases ^18^.

Quantification of tumor mass and clonality by MFC showed increasing bone marrow involvement by clonal B cells from IgM MGUS to smoldering WM and symptomatic WM patients. Additionally, a progressive increase in the proportion of clonal B cells with cytoplasmic expression of the patient-specific light-chain isotype was seen from IgM MGUS to smoldering and symptomatic WM patients. However, despite the progressively higher degree of patient-specific light-chain isotype clonal plasma cells in the bone marrow from IgM MGUS to symptomatic WM, the proportion of bone marrow involvement by clonal plasma cells was similar in all three stages of disease ^18^. IgM levels correlated with the degree of plasma cell clonality but not with the proportion of bone marrow involvement. Clonal B-cell bone marrow infiltration (>10%) and degree of clonality (100%) were highly specific to exclude the diagnosis of IgM MGUS but not to identify patients with WM. The phenotype of clonal plasma cells seen in WM was unrelated to the clonal plasma cells seen in MM ^18^.

Among the cytogenetic findings, chromosome 6q deletion is the most common structural abnormality and is seen in about half of patients with WM, followed by trisomy of chromosome 5 and monosomy 8 ^19^. Fluorescent *in situ* hybridization directed at chromosome 6 confirmed the 6q deletion in 55% of patients with WM but in none of the patients with IgM MGUS ^20^. A genome-wide study of copy number abnormalities (CNAs) and loss of heterozygosity (LOH) using microarray technology identified deletion 6q(23.3–25.3) and +18q(22.1) as the most common structural abnormalities (detected in 16% and 14% of all patients, respectively). Genomic imbalances typically observed in WM (del6q, +18q, trisomy 4 and trisomy 12) were rarely seen in patients with IgM MGUS. The frequency of patients displaying CNAs progressively increased from IgM MGUS (36%) to smoldering (73%) and symptomatic (82%, *P* = 0.03) WM but a similar frequency of LOH was noted in all three disease stages ^21^.

Targeted NGS evaluating the presence of somatic mutations in 11 selected genes ( *MYD88*, *CXCR4*, *ARID1A*, *KMT2D*, *TP53*, *NOTCH2*, *PRDM1*, *CD79b*, *TRAF3*, *TNFAIP3*, and *MYDBBP1A*) in patients with IgM MGUS (n = 56) or WM (n = 63) demonstrated 151 mutations in 74% of the patients. Patients with IgM MGUS harbored a significantly lower number of mutations than patients with WM, conceivably implying that multiple genetic hits are needed for the progression from IgM MGUS to WM. Somatic mutations in MYD88, CXCR4, and KMT2D were more frequently seen in patients with WM (86%, 24%, and 25%, respectively) than in patients with IgM MGUS (46%, 7%, and 5%, respectively), possibly suggesting that mutations in these genes are an earlier event in the pathobiology of IgM MGUS and WM ^22^. A similar proportion of MYD88 ^L265P^ mutation was detected in patients with IgM MGUS (42–54%) or WM (93–97%) when allele-specific polymerase chain reaction was used in an analysis of either bone marrow or peripheral blood samples ^23, 24^. However, no conclusion regarding a driver mutation in those genes can be made. Clonal B cells from bone marrow of patients with IgM MGUS, smoldering WM, and symptomatic WM were evaluated using immunophenotypic protein expression profile (iPEP) and GEP techniques. Early studies demonstrated a distinct molecular signature by GEP between patients with IgM MGUS and those with WM ^25^. However, these findings were not confirmed in later studies involving larger cohorts and a more comprehensive microarray panel ^21, 26^. Virtually no differences were observed in the iPEP or GEP of patients with IgM MGUS compared with patients with WM, implying that the WM clone may already be present in patients with IgM MGUS ^21^.

However, post-translational modifications and epigenetic changes between patients with IgM MGUS and those with WM were not assessed. The iPEP of clonal B cells harboring the MYD88 ^L265P^ mutation was similar to that in MYD88 wild-type cells ^21^. In a comparison of clonal B cells from IgM MGUS and WM with their normal B-cell counterpart (CD22 ^+^ and CD25 ^−^), GEP showed differentially expressed genes related to the interleukin-6 (IL-6), NF-κB, JAK/STAT, PI3K/AKT, inositol tetrakisphosphate (IP4), and 3-phosphoinositide biosynthesis pathways, which are related to cell growth and survival ^21^. The first step in the pathogenesis of MGUS and MM has been hypothesized to be an abnormal response to antigenic stimulation through Toll-like receptors with overexpression of IL 1R leading to increased levels of IL-6 and promotion of an IL-6 autocrine loop ^27^. The next step would be acquisition of genetic abnormalities, probably in a stepwise fashion, leading to the malignant transformation and clonal expansion ^28^. In an attempt to demonstrate common antigenic stimulation in the pathogenies of IgM MGUS and WM, Varettoni *et al*. ^29^ studied the immunoglobulin heavy chain (IGH) variable gene rearrangement repertoire in patients with these conditions. Immunoglobulins, including the monotypic IgM expressed by malignant cells in IgM MGUS and WM, are assembled by somatic recombination of a large number of gene segments in the IGH region and continuously shaped by exposure to exogenous antigens. Finding stereotyped IGH rearrangements could support the hypothesis of an antigenic stimulation as the early event in the development of these diseases. A remarkably biased IGH variable gene usage repertoire was seen in patients with IgM MGUS and WM. A predilection for the IGH variable gene 3 (seen in 83% of patients) in preference to IGH variable genes 1 and 4 was noted. No intra-disease stereotyped clusters were seen. These results imply that the cell of origin of IgM MGUS and WM is a heterogeneous B cell that may have responded to a diversified antigenic stimulation during a physiologic response before its malignant transformation ^29^.

Finding a clear distinction between the malignant cell in patients with IgM MGUS and those with WM has been fundamentally challenging. Although the answer to this question could lie in the fact that these two conditions are merely different stages of the same entity, one cannot overlook a limitation of the studies investigating this question: variability of quality and representativeness of samples analyzed as well as the timing of sampling and handling methods.

## Clinical manifestations of IgM MGUS

Although patients with IgM MGUS have minimal to no bone marrow involvement by LPL and usually low levels of circulating IgM, they may still have symptoms attributable to the IgM properties. Though infrequent, organ damage may result from an IgM paraprotein with autoimmune activity or altered structure prone to tissue deposition ^30^. The term ‘IgM-related disorders’ has been proposed to describe this group of patients which encompass not only IgM MGUS and WM but any underlying condition associated with a monoclonal IgM paraprotein ^9^. Among 377 patients with IgM-related disorders, the most common diagnoses were MGUS (42%), WM (28%), B-cell NHL such as CLL, extranodal marginal zone lymphoma, and splenic marginal zone lymphoma (18%), primary cold agglutinin disease (4%), AL amyloidosis (4%), cryoglobulinemia (2%), and IgM MGUS-associated neuropathy (1%) ^31^.

The peripheral neuropathy associated with IgM MGUS usually presents as a distal, symmetric, and slowly progressive sensorimotor neuropathy with predominantly demyelinating features in the nerve conduction studies. A causal relationship between anti-myelin-associated glycoprotein (MAG) IgM antibodies, identified in half of the patients, and neuropathy has been established. IgM paraprotein directed to other antigens such as GD1b ganglioside, sulphatide, and chondroitin sulphate has also been described, although the pathologic significance is not well understood ^32^. Cold agglutinin disease, leading to extravascular hemolytic anemia after cold exposure caused by IgM binding to red blood cell surface antigens and subsequent complement cascade activation, has been described in patients with IgM MGUS ^33^. Cryoglobulinemia, manifested by acrocyanosis, Raynaud phenomenon, urticaria, peripheral neuropathy, renal failure, or vasculitis resulting from IgM immune-complex precipitation in cold temperatures, has been associated with IgM MGUS as well ^34^. Immune thrombocytopenic purpura and acquired von Willebrand disease are uncommonly seen in IgM MGUS ^35^.

AL amyloidosis, caused by extracellular tissue deposition of misfolded immunoglobulins, is a rare but devastating complication of IgM MGUS ^31^. IgM-related AL amyloidosis less frequently presents with cardiac involvement than non-IgM AL amyloidosis but has a higher prevalence of peripheral neuropathy secondary to the amyloid deposition. Renal failure, seen in up to 68% of the patients, is the most common manifestation ^36^. Schnitzler syndrome is a rare entity characterized by the presence of an IgM paraprotein and a chronic urticarial rash in association with other inflammatory symptoms, lymphadenopathy, hepatosplenomegaly, or bone pain ^37^. The pathophysiology of Schnitzler syndrome is not well understood, but upregulation of the IL-1 pathway plays a pivotal role ^38, 39^. Renal failure can also be seen in IgM MGUS patients as the result of monoclonal protein deposition disease, which includes light-chain, heavy-chain, or light- and heavy-chain deposition disease; proliferative glomerulonephritis; cryoglobulinemic glomerulonephritis; or direct LPL infiltration ^40, 41^.

## Management of IgM MGUS

Owing to the lack of evidence suggesting a survival advantage or prevention/delay of progression to a hematologic malignancy and the concern of treatment-related toxicities, treatment is not recommended for asymptomatic patients with IgM MGUS ^42^. Therefore, a ‘watch and wait’ approach is the preferred choice in asymptomatic patients.

Treatment is indicated for patients presenting with IgM-related disorders. The intensity of treatment should be balanced by the severity of symptoms and potential for short- and long-term sequelae. For minimally symptomatic patients, supportive care may suffice in most cases. For more significant symptoms or risk of irreversible organ damage, treatment directed toward the malignant clone should be considered.

For patients who present with peripheral neuropathy severe and progressive enough to warrant therapy and in whom AL amyloidosis was excluded, a trial of intravenous immunoglobulin (IVIG) could be considered as the initial therapy ^43^. Two randomized, double-blind clinical trials of IVIG versus placebo in patients with IgM-related peripheral neuropathy showed at least partial improvement of symptoms in 28 to 45% of patients. The clinical benefit with IVIG was, however, short-lived ^44, 45^. Rituximab monotherapy has also shown activity in the treatment of IgM-related neuropathy with sustained results. Multiple trials reported at least partial improvement of symptoms in 31 to 86% of patients, and responses lasting more than 36 months were reported in a small prospective single-arm study ^46– 48^. The Eighth International Workshop in Waldenström Macroglobulinemia consensus panel for the diagnosis and management of IgM-associated peripheral neuropathy in patients with MGUS recommends tailoring of the initial therapy on the basis of the presence (or not) of anti-MAG antibodies. For patients with a positive anti-MAG assay, rituximab monotherapy is the preferred first line of therapy, whereas immunosuppressive or immunomodulatory therapy (that is, IVIG, steroids, and plasma exchange) is preferred for patients with a negative anti-MAG assay ^49^. Treatment of patients presenting with IgM-related amyloidosis should follow the standard approach for patients with AL amyloidosis with combination therapy and consideration for autologous stem cell transplant ^50^. Multidrug regimens may also be necessary for the treatment of severe or relapsed/refractory symptoms when an attempt to eradicate the malignant clone is warranted ^30^.

The treatment of Schnitzler syndrome, in contrast, is not directed toward the malignant clone but toward the upregulated cytokine state. Anakinra, an IL-1 receptor antagonist, has been shown to be a very effective therapy in Schnitzler syndrome. In a French multicenter study, all patients (n = 29) treated with anakinra attained a response lasting as long as the patients were followed (median of 36 months) ^51^. Rilonacept, an IL-1 neutralizing fusion protein, and canakinumab, a monoclonal antibody against IL-1 beta, have also demonstrated activity in Schnitzler syndrome ^52, 53^.

## Future perspectives

The understanding of IgM MGUS, as a pre-malignant precursor condition to multiple subtypes of B-cell NHL, is pivotal to comprehend the development of related hematological malignancies, most importantly WM. Available data suggest an aberrant response to a normal antigenic stimulation as the potential initial step in the pathogenesis of IgM MGUS. Additional somatic mutations would be needed to allow the persistence of the abnormal clone, and a multi-hit hypothesis is proposed to explain the progression to WM.

Despite an increased mutation burden seen in patients with WM compared with IgM MGUS, the GEP of clonal B cells is virtually indistinguishable in these two conditions and no driver mutation has been clearly identified to explain why some patients with IgM MGUS progress to WM. Perhaps the answer to this question lies in post-translational or epigenetics changes not yet identified. It is also possible that changes in the bone marrow microenvironment are responsible in part for the progression of IgM MGUS to WM. An effort to prospectively collect samples and information from patients with a variety of pre-malignant conditions, including IgM MGUS, is under way (NCT02269592) and could provide valuable data for future studies.

The role of early treatment of patients with IgM MGUS to prevent or postpone transformation to WM is still unknown. It is unlikely that large clinical trials will be carried out to investigate this question given the rarity of this disease and need for long follow-up. A unique approach using anakinra to induce a chronic disease state in patients with smoldering MM showed promising results but has never been validated in large clinical trials ^54^. One may wonder whether the same concept could be applied to IgM MGUS and WM given the suggested upregulation of the IL-6 pathway seen in these patients and the known association between IL-1 and IL-6.

Many impressive advances have occurred in recent years, shedding light on the pathobiology of IgM MGUS and WM. Our understanding of these conditions is poised to expand in the near future and is likely to favorably impact the outcome of this disease.
